# MiR-410 induces stemness by inhibiting Gsk3β but upregulating β-catenin in non-small cells lung cancer

**DOI:** 10.18632/oncotarget.14529

**Published:** 2017-01-05

**Authors:** Xixian Ke, Yue Yuan, Chenglin Guo, Yan Yang, Qiang Pu, Xueting Hu, Kui Tang, Xinmei Luo, Qianqian Jiang, Xiaolan Su, Lunxu Liu, Wen Zhu, Yuquan Wei

**Affiliations:** ^1^ State Key Laboratory of Biotherapy and Cancer Center/National Collaborative Innovation Center for Biotherapy, West China Hospital, Sichuan University, Chengdu, 610041, Sichuan, China; ^2^ Department of Thoracic Surgery, West China Hospital, Sichuan University, Chengdu, 610041, Sichuan, China

**Keywords:** miR-410, stemness, Wnt/β-catenin, non-small cells lung cancer

## Abstract

Our previous research indicated miR-410 played a critical role in promoting the tumorigenesis and development of NSCLC (non-small cells lung cancer). MiR-410 has been recently reported to be crucial for development and differentiation of embryonic stem cells. But it remains elusive whether miR-410 stimulates the stemness of cancer until now. Herein, we identify miR-410 induces the stemness and is associated with the progression of NSCLC. We demonstrate miR-410 increases the levels of stem cells marker Sox2, Oct4, Nanog, CXCR4 as well as lung cancer stem cells surface marker CD44 and CD166. MiR-410 promotes stem cells-like properties such as proliferation, sphere formation, metastasis and chemoresistance. Moreover, Gsk3β is directly targeted and post-transcriptionally downregulated by miR-410. Also, the expression levels of miR-410 and Gsk3β may be correlated to clinicopathological differentiation in NSCLC tumor specimens. Additionally, we demonstrate miR-410 induces stemness through inhibiting Gsk3β but increasing Sox2, Oct4, Nanog and CXCR4, which binds to β-catenin signaling. In conclusion, our findings identify the miR-410/Gsk3β/β-catenin signaling axis is a novel molecular circuit in inducing stemness of NSCLC.

## INTRODUCTION

Despite drastic treatment strategies, including radiotherapy and chemotherapy, the 5-year survival rate of NSCLC remained a low level of 15% [1]. Postoperative metastasis and chemoresistance accounted for its treatment failure and relapse. Emerging evidences suggested the enriched existence of tumor initiating cells, also called cancer stem cells (CSCs), with the potency of self-renewal, differentiation and high oncogenicity, primarily accounted for the metastasis, reoccurrence, and chemoresistance in many tumors [2–4]. Cancer was acknowledged to originate from CSCs [5], and many tumors (including lung cancer [6, 7]) might progress because of CSCs. However, little was known about the regulating mechanism of lung cancer stemness. Therefore, revealing those regulating mechanisms of lung cancer stemness would contribute to uncovering the molecular mechanism of lung cancer tumorigenesis and development.

Recently, multiple studies had highlighted that miRNA deregulations were fundamental in the regulation of CSCs properties such as self-renewal, capacity to generate a progeny of differentiated cells, chemoresistance and maintenance of stemness [8]. Quantities of miRNAs had been discovered to modulate stemness in several of tumors, whereas only a spot of miRNAs were correlated to stemness in lung cancer [9–11]. MiR-410, a member of the largest known miRNA cluster miR-379-410 [12], played a critical role in distinct tumors (such as pancreatic cancer [13], breast cancer [14], liver and colorectal tumors [15], etc.) via promoting or inhibiting cells proliferation, apoptosis, invasion, migration and angiogenesis. Recently, we also demonstrated miR-410 was significantly upregulated in NSCLC cells and tumor tissues, and acted as oncogene which might be correlated to Wnt/β-catenin pathway [16]. Our previous research indicated miR-410 played a critical role in promoting the tumorigenesis and development of NSCLC. Whereas, the molecular mechanism of miR-410 on this progression was still little illuminated.

Furthermore, miR-410 was recently proposed to be involved in the development and differentiation of embryo or embryonic stem cells [17–18]. Moreover, overexpression of miR-410 and miR-433 rescued myogenic differentiation in Mef2a-deficient myoblasts through repressing sFRPs, indicating miR-410-mediated activation of WNT signaling was a prerequisite qualification for muscle accurate regeneration [19]. Actually, Wnt/β-catenin signaling was not only correlated to tumor cells proliferation, invasion and migration, differentiation, but also to cancer stem cells in maintaining stemness, self-renewal or differentiation [20–22]. Activating of Wnt/β-catenin signaling promoted the transformation of G1 to S stage and stem-like properties (such as capacities of proliferation, clonogenicity, metastasis and chemoresistance etc.) in lung cancer A549 cells [23]. However, the role of Wnt/β-catenin signaling on promoting or inhibiting stemness was still poorly elucidated in lung cancer.

Additionally, in our preliminary experiments, we found miR-410 increased the expressions of stem cells marker Sox2 and Oct4 in A549 or H1299 cells after transfecting with miR-410 mimics/NC, while decreased their expressions in those cells after transfecting with miR-410 inhibitors/NC (Data not shown). In view of our previous research that miR-410 promoted the tumorigenesis and development of NSCLC, and others’ researches, we hypothesized miR-410 might promote the progression of NSCLC by influencing stemness of NSCLC. Therefore, in present study, we aimed to further explore the function and molecular mechanism of miR-410 in promoting the progression of NSCLC via inducing stemness. We firstly demonstrated miR-410 induced stem cells-like capacities in NSCLC cells. Then, we found that Gsk3β was a direct target of miR-410 in inducing stemness of NSCLC. Finally, our findings unraveled the miR-410/Gsk3β/β-catenin signaling axis was a novel molecular circuit in inducing stemness of NSCLC.

## RESULTS

### MiR-410 increased the expressions of stem cells markers Nanog, Sox2, Oct4, CXCR4 and lung cancer stem cells surface marker CD44 and CD166 in NSCLC cells

**Figure 1:**
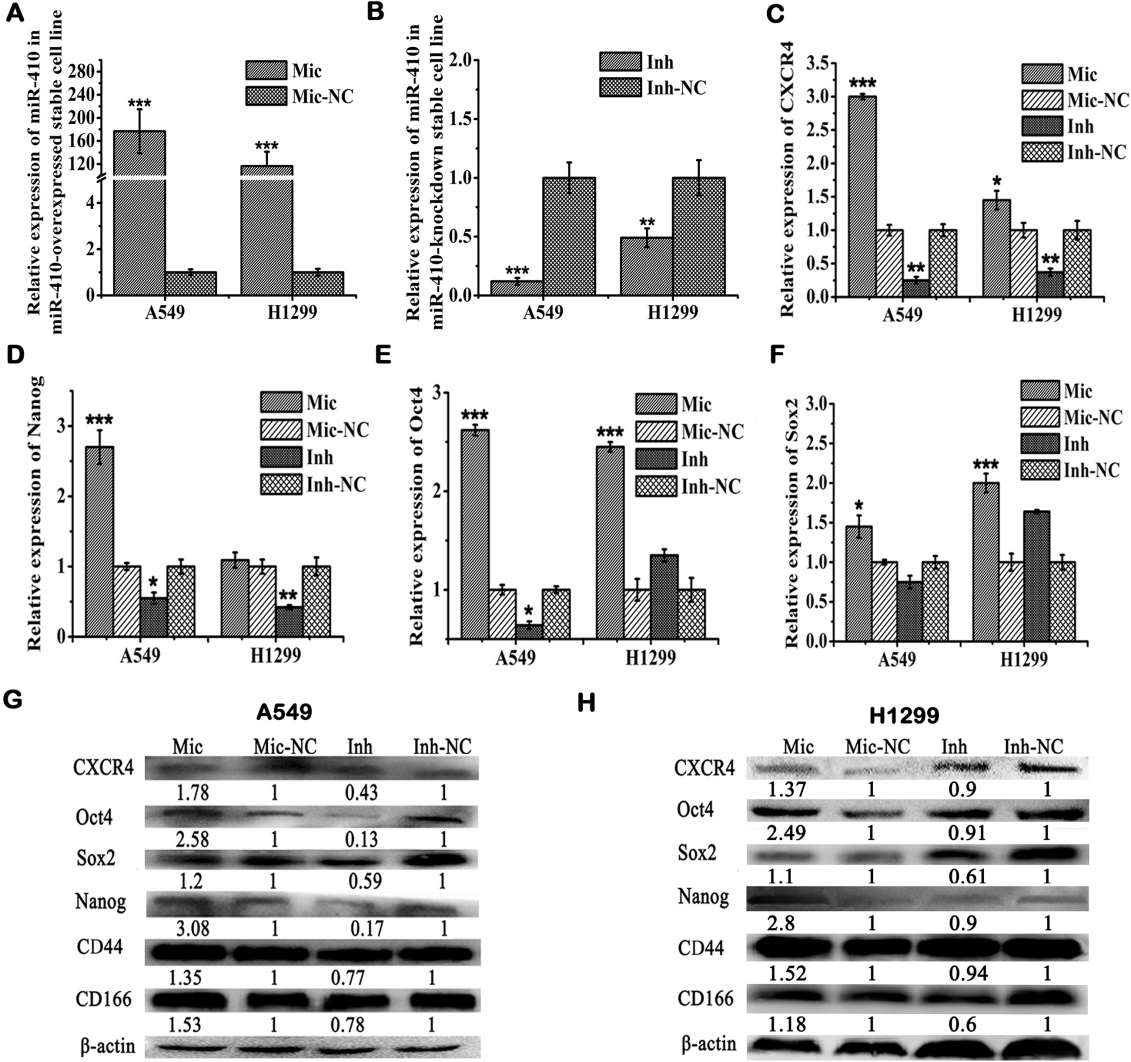
MiR-410 increased the expressions of stem cells markers Oct4, Sox2, Nanog, CXCR4 and cells surface markers CD44 and CD166 in NSCLC cells (**A**) and (**B**) Expressions of miR-410 were detected by qRT-PCR in miR-410 overexpression (A) or knock-down (B) stable cells derived from A549 and H1299 cells. (**C**–**E**) and (**F**) Expressions of CXCR4 C., Nanog D., Oct4 (E) and Sox2 (F) were detected by qRT-PCR respectively in miR-410 overexpression or knock-down stable cells derived from A549 and H1299 cells. (**G**) and (**H**) Protein expression of CXCR4, Nanog, Oct4, Sox2, and stem cells surface markers CD44 and CD166 were detected by western blotting respectively in miR-410 overexpression or knock-down stable cells derived from A549 (G) and H1299 (H) cells. Mic and Mic-NC, miR-410 overexpression stable cells and its matched NC control stable cells; Inh and Inh-NC, miR-410 knock-down stable cells and its matched NC control stable cells. *P < 0.05; **P < 0.01; ***P < 0.001. *compared with the relative NC control.

In order to explore the effects of miR-410 on regulating stemness of NSCLC, we firstly established miR-410 overexpression and knock-down stable cells as well as NC control cells using A549 and H1299 cells by infecting with miR-410 overexpression or inhibiting lentivirus particles. The level of miR-410 in overexpression stable A549 or H1299 cells was increased by 176.78 or 116.46 fold respectively (Figure 1A), versus that was decreased by 0.12 or 0.49 fold respectively in knock-down stable A549 or H1299 cells compared with their respective NC control cells detected by qRT-PCR (Figure 1B).

Next, we checked the effects of miR-410 on the expressions of stem cells markers such as Nanog, Sox2, Oct4 and CXCR4. The mRNA levels of CXCR4, Nanog, Oct4, and Sox2 were remarkably increased in miR-410 overexpression stable A549 cells versus decreased in miR-410 knock-down stable A549 cells compared with their respective NC control cells by qRT-PCR (Figure 1C–1E and 1F). The mRNA levels of CXCR4, Oct4, and Sox2 were remarkably increased in miR-410 overexpression stable H1299 cells versus that of CXCR4 and Nanog were obviously decreased in miR-410 knock-down stable H1299 cells compared with their respective NC control cells by qRT-PCR (Figure 1C–1E and 1F). Similarly, the protein levels of Nanog, Sox2, Oct4 and CXCR4 were also apparently increased in miR-410 overexpression stable A549 (Figure 1G) or H1299 (Figure 1H) cells versus decreased in miR-410 knock-down stable A549 (Figure 1G) or H1299 (Figure 1H) cells compared with their respective NC control cells detected by Western blotting.

Several researchers recently reported CD44 and CD166 were stem cells markers of lung cancer [24–27]. Therefore, we also detected the effects of miR-410 on the expressions of CD44 and CD166 in lung cancer cells. The protein level of CD44 or CD166 was significantly elevated in miR-410 overexpression stable A549 (Figure 1G) or H1299 (Figure 1H) cells versus that was decreased in miR-410 knock-down stable A549 (Figure 1G) or H1299 (Figure 1H) cells compared with their respective NC control cells by Western blotting. Taken together, all of these data gave a hint that miR-410 impelled the producing of stemness in NSCLC cells.

### MiR-410 augmented sphere formation and proliferation abilities of NSCLC

**Figure 2:**
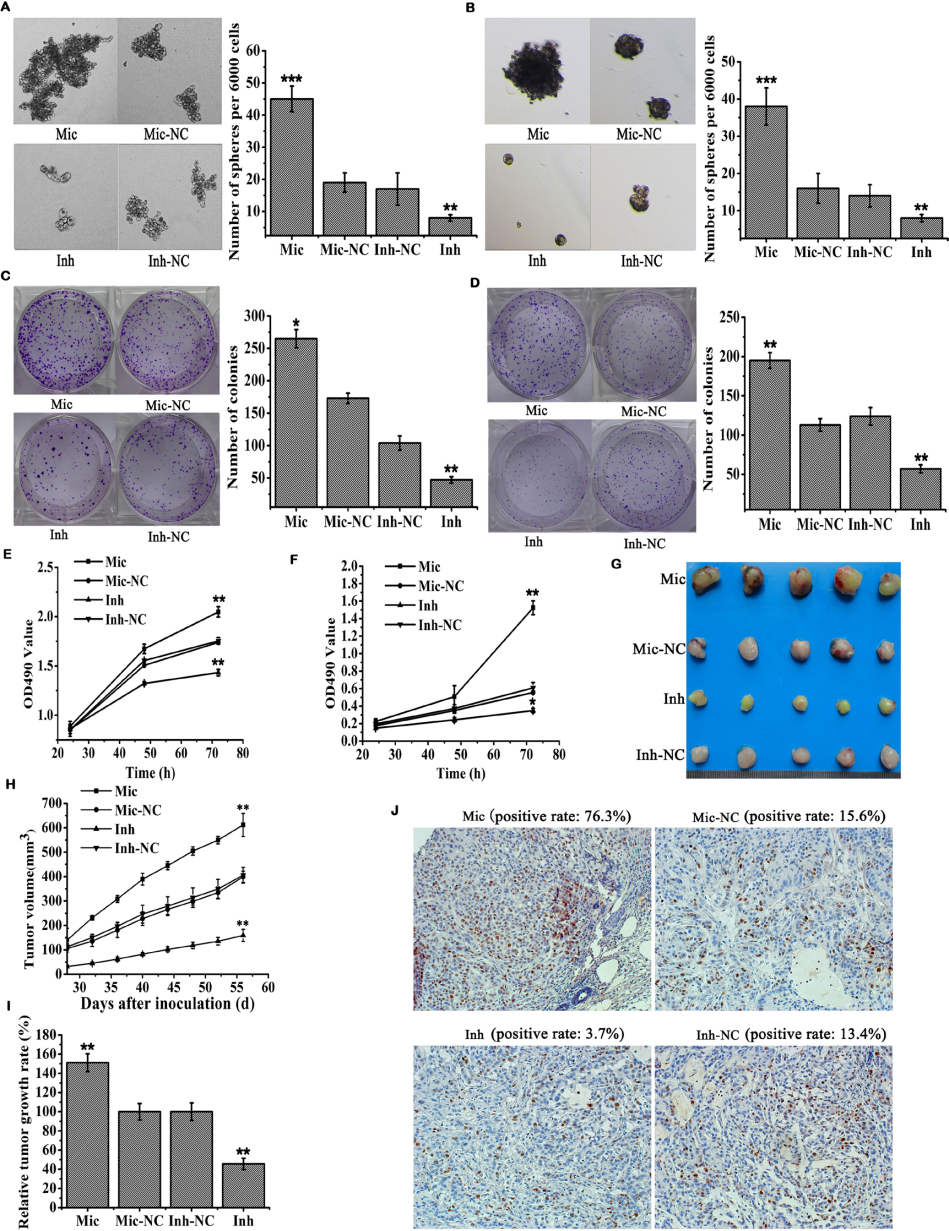
MiR-410 augmented sphere formation and proliferation abilities of NSCLC (**A** and **B**) Sphere formation assays of miR-410 stable overexpressed or knock-down cells derived from A549 (A) and H1299 (B) cells. Mic and Mic-NC, miR-410 overexpression stable cells and its matched NC control stable cells; Inh and Inh-NC, miR-410 knock-down stable cells and its matched NC control stable cells. (**C** and **D**) Colony formation assays of miR-410 overexpression or knock-down stable cells derived from A549 C. and H1299 (D) cells. Mic and Mic-NC, miR-410 overexpression stable cells and its matched NC control stable cells; Inh and Inh-NC, miR-410 knock-down stable cells and its matched NC control stable cells. (**E** and **F**) MTT assays of miR-410 overexpression or knock-down stable cells derived from A549 (E) and H1299 (F) cells. Mic and Mic-NC, miR-410 overexpression stable cells and its matched NC control stable cells; Inh and Inh-NC, miR-410 knock-down stable cells and its matched NC control stable cells. (**G**–**I**) and (**J**) Images (G) the growth curves (H) the relative growth rate (I) and Ki67 immunohistochemical staining (J) of subcutaneous tumor. Mic and Mic-NC, mice treated with miR-410 overexpression stable cells and its matched NC control stable cells; Inh and Inh-NC, mice treated with miR-410 knock-down stable cells and its matched NC control stable cells. *P < 0.05; **P < 0.01; ***P < 0.001. *compared with the relative NC control.

We had initially proved miR-410 increased the expressions of stemness markers in A549 and H1299 cells. In order to further confirm the role of miR-410 in promoting stemness, we next determined the functional properties of miR-410 in increasing stem cells-like characteristics. Sphere forming assay displayed miR-410 overexpression stable A549 (Figure 2A) or H1299 (Figure 2B) cells formed much more and bigger spheres versus miR-410 knock-down stable A549 (Figure 2A) or H1299 (Figure 2B) cells formed less and smaller spheres compared with their respective NC control cells. Clonogenic assay indicated the colony numbers of miR-410 overexpression stable A549 (Figure 2C) or H1299 (Figure 2D) cells were larger versus that were smaller in miR-410 knock-down stable A549 (Figure 2C) or H1299 (Figure 2D) cells compared with their respective NC control cells.

*In vitro* MTT assay showed cells proliferation was observably promoted in miR-410 overexpression stable A549 (Figure 2E) or H1299 (Figure 2F) cells versus that was markedly inhibited in miR-410 knock-down stable A549 (Figure 2E) or H1299 (Figure 2F) cells compared with their respective NC control cells. Moreover, the subcutaneous lung tumor mouse model was established to check the effect of miR-410 on cells proliferation *in vivo*. The subcutaneous xenotransplanted tumors in nude mice were sacrificed about 8 weeks after subcutaneously injecting with miR-410 overexpression stable cells or NC control cells (Figure 2G). The growth curve of subcutaneous xenotransplanted tumor volume was shown in Figure 2H. And, the relative tumor growth rate in nude mice injected with miR-410 overexpression stable cells was much faster versus that was slower in nude mice injected with miR-410 knock-down stable cells compared with nude mice injected with their respective control cells (Figure 2I). Ki67 immunohistochemical staining of subcutaneous xenotransplanted tumor tissues further confirmed the positive rate of Ki67 was greater in tumor tissues of mice injected with miR-410 overexpression stable cells versus that was smaller in tumor tissues of mice injected with miR-410 knock-down stable cells compared with mice injected with their respective control cells (Figure 2J). All of these results demonstrated miR-410 could strengthen the stem cells-like characteristics such as promoting proliferation and sphere formation of NSCLC *in vitro and in vivo*.

### MiR-410 promoted metastasis and cisplatin-resistance of NSCLC

**Figure 3:**
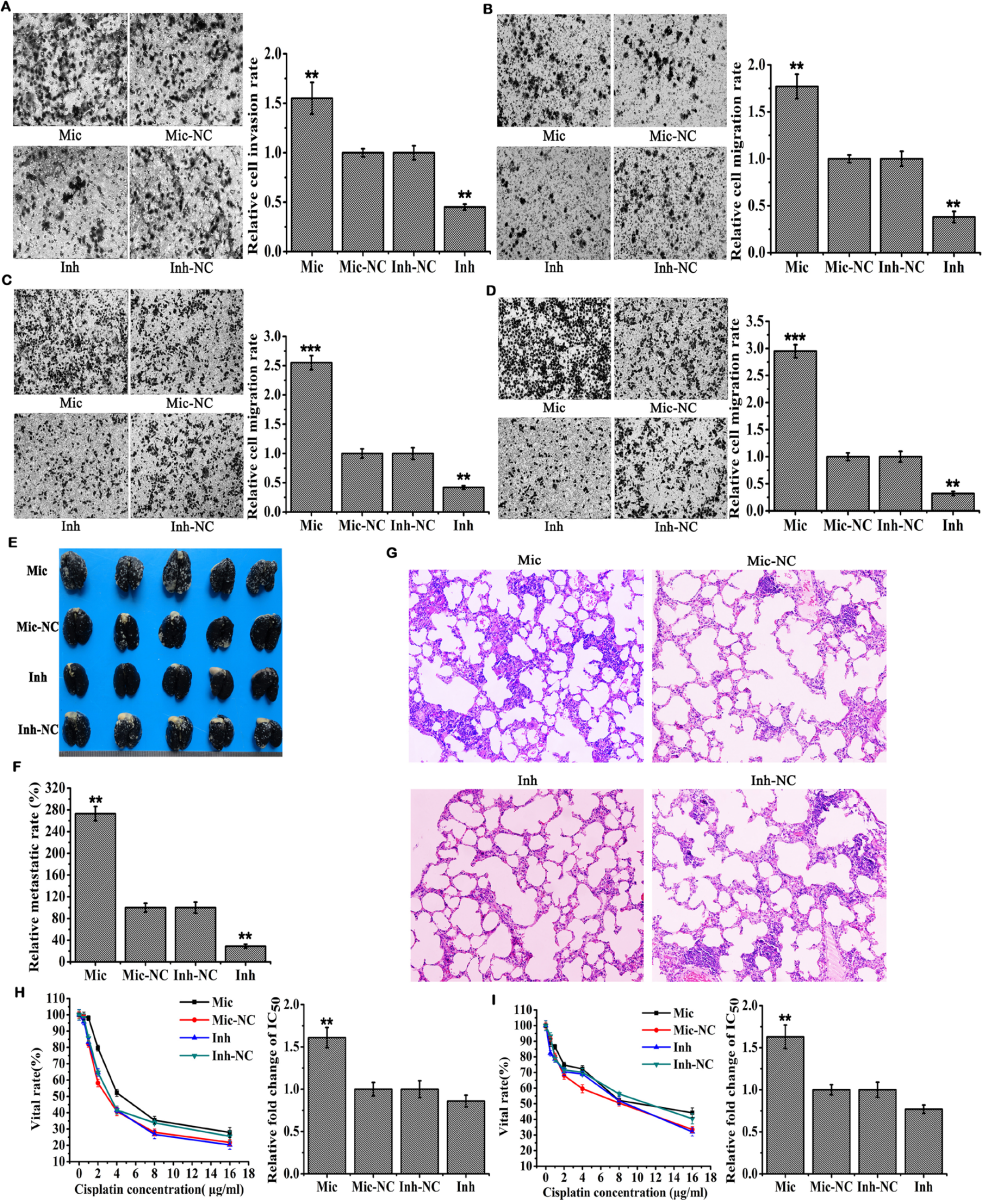
MiR-410 promoted metastasis and drug resistance of NSCLC (**A**) and (**B**) In vitro Transwell assays of miR-410 overexpression or knock-down stable cells derived from A549 A. and H1299 B. cells. Mic and Mic-NC, miR-410 overexpression stable cells and its matched NC control stable cells; Inh and Inh-NC, miR-410 knock-down stable cells and its matched NC control stable cells. (**C**) and (**D**) In vitro Millicells assays of miR-410 overexpression or knock-down stable cells derived from A549 C. and H1299 D. cells. Mic and Mic-NC, miR-410 overexpression stable cells and its matched NC control stable cells; Inh and Inh-NC, miR-410 knock-down stable cells and its matched NC control stable cells. (**E**, **F**) and (**G**) Images of metastatic nodules in the lungs of mice (E) relative lung metastatic rate of each treatment group (F) and images of H&E staining with lung tissues of nude mice (G). Mic and Mic-NC, mice treated with miR-410 overexpression stable cells and its matched NC control stable cells; Inh and Inh-NC, mice treated with miR-410 knock-down stable cells and its matched NC control stable cells. (**H**) and (**I**) Cells vital rates of miR-410 overexpression or knock-down stable cells derived from A549 H. and H1299 I. cells were resistant to Cisplatin. IC50 was half maximal inhibitory concentration calculated by SPSS software. Mic and Mic-NC, miR-410 overexpression stable cells and its matched NC control stable cells ; Inh and Inh-NC, miR-410 knock-down stable cells and its matched NC control stable cells. *P < 0.05; **P < 0.01; ***P < 0.001. *compared with the relative NC control.

We further tested the effects of miR-410 on stem cells-like characteristics of metastasis and drug resistance. *In vitro* Transwell assay indicated cells invasion was significantly promoted in miR-410 overexpression stable A549 (Figure 3A) or H1299 (Figure 3B) cells versus that was apparently inhibited in miR-410 knock-down stable A549 (Figure 3A) or H1299 (Figure 3B) cells compared with their respective NC control cells. Similarly, *in vitro* Millicells assay showed cells migration was markedly enhanced in miR-410 overexpression stable A549 (Figure 3C) or H1299 (Figure 3D) cells versus that was obviously impaired in miR-410 knock-down stable A549 (Figure 3C) or H1299 (Figure 3D) cells compared with their respective NC control cells.

Furthermore, lung metastasis mouse model was established to detect the effect of miR-410 on metastasis *in vivo*. We observed that the numbers of tumor metastasis nodules (white dots) in the lungs of mice injected with miR-410 overexpression stable cells were more and bigger versus those were less and smaller in lungs of mice injected with miR-410 knock-down stable cells compared with mice injected with their respective control cells (Figure 3E and 3F). Consistently, much more and bigger nodules were found in lung tissue sections from mice injected with miR-410 overexpression stable cells by H&E staining compared with mice injected with their respective control cells. But less and smaller nodules were found in lung tissue sections from mice injected with miR-410 knock-down stable cells by H&E staining compared with mice injected with their respective control cells (Figure 3G). These results further demonstrated miR-410 promoted metastasis of NSCLC *in vivo*.

Cisplatin was a widespread therapeutic drug for lung cancer. It was used to investigate the effect of miR-410 on drug resistance in this study. We found that IC_50_ of miR-410 overexpression stable A549 (IC_50_: Mic vs Mic-NC = 5.8 μM vs 3.61 μM) (Figure 3H) or H1299 (IC_50_: Mic vs Mic-NC = 11.11 μM vs 6.83 μM) cells (Figure 3I) was 1.61 or 1.63 fold respectively versus that was 0.86 or 0.77 fold respectively in miR-410 knock-down stable A549 (IC_50_: Inh vs Inh-NC = 3.68 μM vs 4.28 μM) (Figure 3H) or H1299 (IC_50_: Inh vs Inh-NC = 7.83 μM vs 10.16 μM) (Figure 3I) cells compared with their respective NC control cells. These results indicated miR-410 increased the resistance of NSCLC to cisplatin. To conclude, all of these findings affirmed miR-410 strengthened the stem cells-like characteristics such as metastasis and drug resistance in NSCLC.

### Gsk3β was a direct target of miR-410

**Figure 4:**
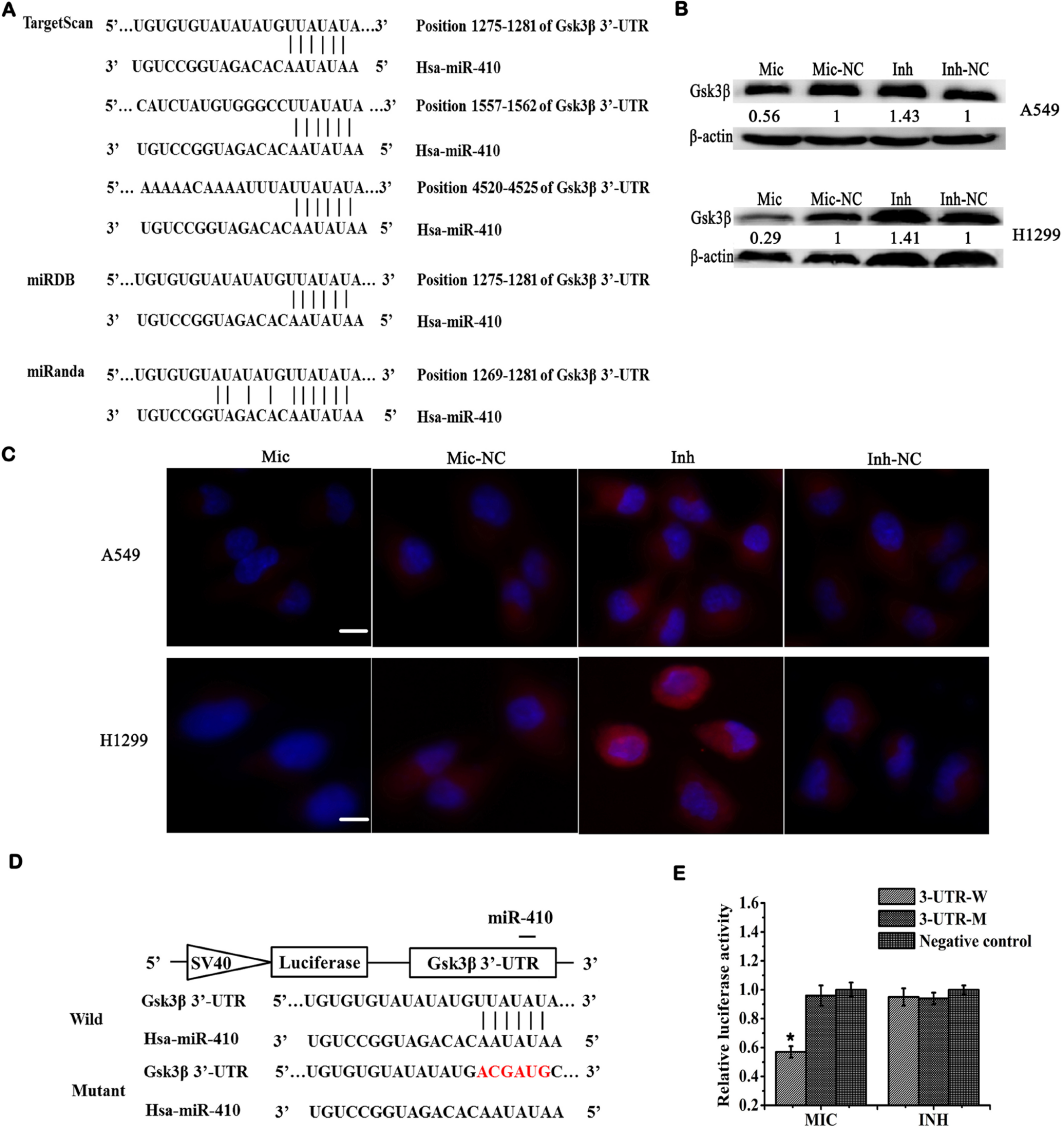
Gsk3β was a direct target of miR-410 (**A**) Target prediction of miR-410 by bioinformatics software TargetScan, miRDB and miRanda. (**B**) Expressions of Gsk3β were detected by Western blotting in miR-410 overexpression or knock-down stable cells derived from A549 and H1299 cells. (**C**) Immunofluorecent assays of Gsk3β in miR-410 overexpression or knock-down stable cells derived from A549 and H1299 cells. Red: Gsk3β; Blue: nucleus. (**D**) Schema chart of miR-410 binding to 3′UTR sequences of Gsk3β as well as wild and mutant binding sites sequence information. (**E**) Luciferase reporter assays were performed to verify the miR-410 binding to the 3′UTR of Gsk3β. The luciferase activity was detected in miR-410 overexpression or knock-down stable cells derived from A549 cells after transfecting with luciferase reporter plasmids (pmirGLO-Gsk3β-3′UTR-wild, 3-UTR-W; pmirGLO-Gsk3β- 3′UTR-mutant, 3-UTR-M) or pmirGLO plasmid. Mic and Mic-NC, miR-410 overexpression stable cells and its matched NC control stable cells ; Inh and Inh-NC, miR-410 knock-down stable cells and its matched NC control stable cells. *P < 0.05; **P < 0.01; ***P < 0.001. *compared with the relative NC control.

In light of that we had proved miR-410 induced stemness of NSCLC, we next aimed to explore the molecular mechanism of miR-410 inducing stemness. Three algorithms (TargetScan, miRanda and miRDB) were used to predict potential target of miR-410. Gsk3β was predicted to be a promising target (Figure 4A). Take consideration of our previous results that miR-410 decreased Gsk3β expression in NSCLC [16], we strategically selected Gsk3β as a candidate target of miR-410. To confirm it, we first investigated its expression level in miR-410 overexpression and knock-down stable A549 or H1299 cells. And we found that its protein level was greatly decreased in miR-410 overexpression stable A549 or H1299 cells, but increased in miR-410 knock-down stable A549 or H1299 cells compared with their respective NC control cells (Figure 4B). However, the mRNA level of Gsk3β made no difference in miR-410 overexpression or knock-down stable A549 and H1299 cells (data not shown). Immunofluorescence staining of Gsk3β also displayed the fluorescence intensity of Gsk3β in miR-410 overexpression stable A549 or H1299 cells spheres were evidently stronger versus that was notably weaker in miR-410 knock-down stable A549 or H1299 cells spheres compared with their respective NC control cells (Figure 4C).

We further applied luciferase reporter assay to verify target relationship of miR-410 and Gsk3β. Luciferase reporter plasmid containing the wild-type or mutant 3′ UTR sequences of Gsk3β was constructed (Figure 4D). After transfecting with wild-type reporter plasmid 3-UTR-W, the relative luciferase activity was significantly reduced in miR-410 overexpression stable cells compared with NC control cells (Figure 4E). In contrast, the relative luciferase activity was not affected in miR-410 overexpression stable cells compared with NC control cells after transfecting with the mutant reporter plasmid 3-UTR-M (Figure 4E). Whereas, the relative luciferase activities showed no significant difference in miR-410 knock-down stable cells compared with NC control cells after transfecting with wild-type reporter plasmid 3-UTR-W or the mutant reporter plasmid 3-UTR-M (Figure 4E). These data confirmed miR-410 directly targeted the 3′-UTR of Gsk3β and Gsk3β was post-transcriptionally down-regulated by miR-410 in NSCLC.

### MiR-410 induced stemness via down-regulating Gsk3β but increasing β-catenin expression

**Figure 5:**
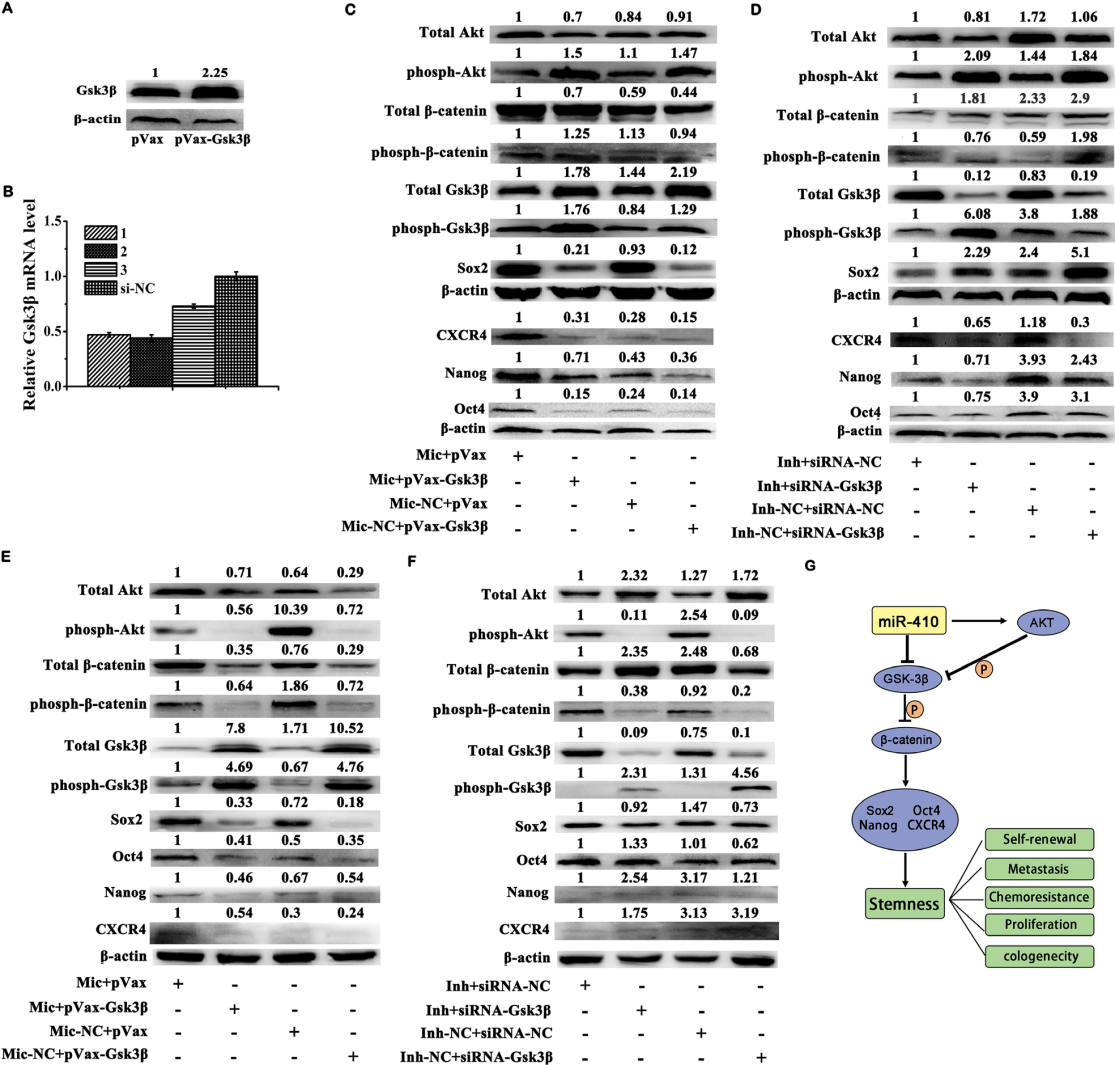
MiR-410 induced stemness via down-regulating Gsk3β but increasing β-catenin expression (**A**) Expressions of Gsk3β were detected by Western blotting in 293T cells after transfecting with pVax-Gsk3β or pVax plasmids. (**B**) Expressions of Gsk3β were detected by qRT-PCR in 293T cells after transfecting with siRNA-Gsk3β (1#, 2# and 3#) or siRNA-NC. (**C**) and (**D**) Expressions of total Akt, phosph-Akt, total β-catenin, phosph-β-catenin, total Gsk3β, phosph-Gsk3β, Oct4, Sox2, Nanog and CXCR4 were detected by Western blotting in miR-410 overexpression stable cells after transfecting with pVax-Gsk3β/pVax plasmids (C) or in miR-410 knock-down stable cells after transfecting with siRNA-Gsk3β/siRNA-NC (D) derived from A549 cells. (**E**) and (**F**) Expressions of total Akt, phosph-Akt, total β-catenin, phosph-β-catenin, total Gsk3β, phosph-Gsk3β, Oct4, Sox2, Nanog and CXCR4 were detected by Western blotting in miR-410 overexpression stable cells after transfecting with pVax-Gsk3β/pVax plasmids E., or in miR-410 knock-down stable cells after transfecting with siRNA-Gsk3β/ siRNA-NC F. derived from H1299 cells. G. The schema chart depicted miR-410/Akt/Gsk3β/β-catenin signaling axis modulated the stemness of NSCLC. Mic and Mic-NC, miR-410 overexpression stable cells and its matched NC control stable cells; Inh and Inh-NC, miR-410 knock-down stable cells and its matched NC control stable cells. *P < 0.05; **P < 0.01; ***P < 0.001. *compared with the relative NC control.

In order to demonstrate whether miR-410 inducing stemness was mediated by down-regulating Gsk3β and stimulating Wnt/β-catenin pathway, we checked the expressions of Gsk3β and β-catenin as well as Nanog, Sox2, Oct4 and CXCR4 in miR-410 stable overexpressed A549 or H1299 cells after transfecting with pVax-Gsk3β/pVax, or in miR-410 stable knock-down A549 or H1299 cells after transfecting with siRNA-Gsk3β/siRNA-NC. In light of that Gsk3β inactivated by Akt at a serine 9 phosphorylation caused an accumulation of β-catenin in the cytoplasm, and then facilitated the translocation of β-catenin to the nucleus in which it activated the expressions of downstream targets [28], we also examined the expression of Akt in those conditions. The protein level of Gsk3β in 293T cells was 2.25 fold after transfecting with pVax-Gsk3β compared with that transfecting with pVax plasmid (Figure 5A). The expressions of Gsk3β in 293T cells were reduced by 53%, 56% and 27% respectively after transfecting with three designed siRNA sequences of Gsk3β (Named 1#, 2# and 3# respectively) compared with that transfecting with siRNA-NC (Figure 5B). Thus, 2# siRNA-Gsk3β was applied in the following transfections.

The up-regulations of total Akt, total β-catenin, phosph-Gsk3β, Nanog, Sox2, Oct4 and CXCR4 were partly reduced in miR-410 overexpression stable A549 (Figure 5C) or H1299 (Figure 5E) cells after transfecting with pVax-Gsk3β compared with pVax. The expressions of phosph-Akt, phosph-β-catenin (Ser33/37/Thr41), total Gsk3β, Nanog, Sox2, Oct4 and CXCR4 were reduced in miR-410 overexpression stable A549 (Figure 5C) or H1299 (Figure 5E) NC control cells after transfecting with pVax-Gsk3β compared with pVax.

Whereas, the down-regulations of total Akt, total β-catenin, phosph-Gsk3β, Nanog, Sox2, Oct4 and CXCR4 were partly reduced in miR-410 knock-down stable A549 (Figure 5D) or H1299 (Figure 5F) cells after transfecting with siRNA-Gsk3β compared with siRNA-NC. The expressions of phosph-Akt, phosph-β-catenin (Ser33/37/Thr41), total Gsk3β, Nanog, Sox2, Oct4 and CXCR4 were increased in miR-410 knock-down stable A549 (Figure 5D) or H1299 (Figure 5F) NC control cells after transfecting with siRNA-Gsk3β compared with that transfecting with siRNA-NC. These data indicated that miR-410 induced stemness of NSCLC cells via down-regulating Gsk3β but up-regulating β-catenin and Nanog, Sox2, Oct4 and CXCR4. The schema chart of miR-410/Akt/Gsk3β/β-catenin signaling axis inducing the stemness of NSCLC was depicted in Figure 5G.

### The levels of miR-410 and Gsk3β were correlated to clinicopathological differentiation in NSCLC tumor specimens

**Figure 6:**
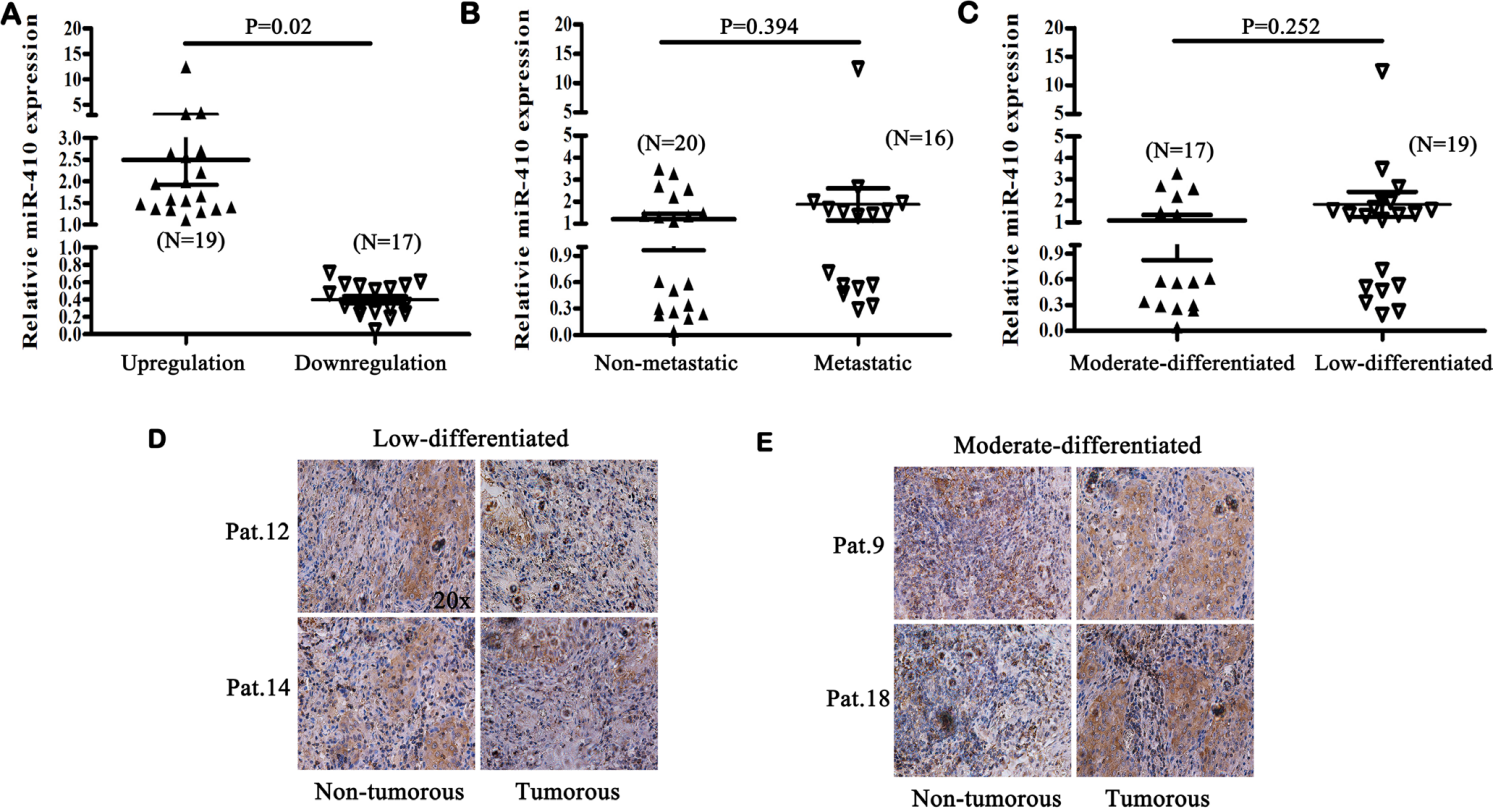
The levels of miR-410 and Gsk3β were correlated to clinicopathological differentiation in NSCLC tumor specimens (**A**) The mean relative level of miR-410 in miR-410high (N = 19) or miR-410low (N = 17) tumor tissues. (**B**) The mean relative level of miR-410 in metastatic (N = 16) or non-metastatic (N=20) tumor tissues. C. The mean relative level of miR-410 in low-differentiated (N = 19) or moderate-differentiated (N = 17) tumor tissues. (**D**) and (**E**) Representative pictures of immunohistochemical staining of Gsk3β in human low-differentiated D. or moderate -differentiated E. tumor tissues and adjacent normal tissues of NSCLC. Statistical analysis was performed using Student's t-test.

In the following, we further tested the reverse expression relationship between miR-410 and Gsk3β, as well as their correlation with clinicopathological characteristics (metastasis and differentiation) in 36 pairs of human NSCLC tumor tissues and adjacent normal tissues. We found miR-410 was significantly overexpressed in 19 tumor tissues while downregulated in 17 tumor tissues compared with their respective non-tumorous tissues, and the relative mean expressions of miR-410 were significantly higher in the miR-410^high^ tumor tissues than those in the miR-410^low^ tumor tissues (*P* = 0.02) (Table 1, Figure 6A). The relative mean levels of miR-410 were not significantly higher in metastatic tumor tissues than that in non-metastatic tumor tissues (*P* = 0.394) (Figure 6B). And, the miR-410 levels were not statistically correlated to the *in vivo* metastasis in tumor tissues (*P* = 0.709) (Table 2).

**Table 1:** Patient clinical features and miR-410 expression profile

Pat. No.	Gender	Age	Differentiation	Metastatic or non-metastatic	Histological grade	Clinical stage	Relative miR-410 Expression
1	M	67	Low	N	S	IA	1.36
2	M	51	Medium	N	A	IIA	0.61
3	F	40	Low	Y	S	IA	0.53
4	M	53	Low	Y	A	IIIA	0.71
5	M	65	Medium	N	S	IB	2.21
6	F	70	Low	Y	A	IA	0.47
7	F	56	Medium	N	A	IB	2.57
8	M	57	Low	Y	A	IIA	1.66
9	M	47	Medium	Y	S	IIA	0.29
10	F	50	Low	Y	S	IA	12.43
11	M	64	Low	N	A	IB	0.23
12	M	63	Low	N	S	IV	3.49
13	M	57	Low	N	A	IB	1.30
14	M	61	Low	Y	S	IV	1.95
15	M	67	Low	N	A	IA	0.19
16	M	69	Medium	N	S	IA	1.37
17	M	51	Low	Y	A	IIA	0.33
18	M	58	Medium	N	S	IIA	0.26
19	M	69	Low	N	S	IA	1.11
20	F	60	Medium	N	A	IA	1.48
21	M	57	Low	N	S	IB	0.51
22	F	48	Medium	N	A	IB	3.28
23	F	61	Low	Y	A	IIIA	1.58
24	M	57	Low	Y	A	IV	1.41
25	F	73	Medium	N	A	IV	0.044
26	F	52	Medium	N	A	IA	0.58
27	M	59	Medium	N	S	IA	0.24
28	M	52	Low	Y	S	IIB	1.99
29	M	53	Medium	Y	A	IIB	0.56
30	M	63	Medium	N	A	IA	2.7
31	M	66	Low	Y	A	IIA	2.64
32	F	68	Medium	Y	A	IIB	0.57
33	M	73	Low	Y	A	IIB	1.56
34	F	50	Medium	Y	A	IIA	1.35
35	F	41	Medium	N	A	IIA	0.3
36	F	63	Medium	N	A	IA	0.34

Note: M, male; F, female; A, adenocarcinoma; S, squamous cell carcinoma; L, large cell carcinoma; Y, yes; N, no. Relative expression of miR-410 was performed by the 2^−ΔΔCt^ method with adjacent non-tumorous lung tissues as a calibrator. Data show the means from independent analyses. Every independent analysis by Real - time PCR was carried out immediately after the RNA extraction and reverse transcribed. ΔCt obtained from real-time PCR was subjected to paired *t*-test (ΔCt = Ct_miR-410_−Ct_U6_).

**Table 2:** Statistical analysis of miR-410 expression with clinicopathological characteristics in human NSCLC tumor specimens

MiR-410 expression	Metastasis		*P* value	Differentiation		*P* value
	Yes	No		Low	Medium	
Downregulation	7 (7/16)	10 (10/20)	0.709	6 (6/19)	11 (11/17)	0.047*
Upregulation	9 (9/16)	10 (10/20)	0.709	13 (13/19)	6 (6/17)	0.047*

*P* by Χ^2^ test; *P < 0.05.

The relative mean levels of miR-410 were not significantly higher in low-differentiated tumor tissues than that in moderate-differentiated tumor tissues (*P* = 0.252) (Figure 6C). However, miR-410^low^ apparently existed in 11 of 17 moderate-differentiated tumor tissues versus that existed in 6 of 19 low-differentiated tumor tissues (*P* = 0.047), and miR-410^high^ significantly existed in 13 of 19 low-differentiated tumor tissues versus that existed in 6 of 17 moderate-differentiated tumor tissues (*P* = 0.047) (Table 2). Additionally, the positive staining of Gsk3β detected by immunohistochemistry was apparently less in 8 of 13 miR-410^high^ low-differentiated (Figure 6D) versus that was more in 7 of 11 miR-410^low^ moderate-differentiated tumor tissues (Figure 6E), compared with their respective non-cancerous tissues. These results further cued that cells with high level of miR-410 but low expression of Gsk3β existed in human NSCLC tissues. Also, the high level of miR-410 and low expression of Gsk3β might be correlated to clinicopathological differentiation in NSCLC tumor specimens.

## DISCUSSION

In our previous study, we reported miR-410 acted as oncogene which might be correlated to Wnt/β-catenin pathway. However, the molecular mechanism of miR-410 on the tumorigenesis and development of NSCLC was still little understood. In present study, we firstly revealed miR-410 promoted the progression of NSCLC through inducing stemness via inhibiting Gsk3β but increasing β-catenin expression. MiR-410 elevated the expressions of stem cells markers such as Oct4, Sox2, Nanog, CXCR4 and putative lung cancer stem cells surface marker CD44 and CD166. Mir-410 also promoted stem-like characteristics such as proliferation, sphere formation, metastasis, chemoresistance, etc. Moreover, Gsk3β was directly targeted and post-transcriptionally downregulated by miR-410. We also demonstrated that down-regulation of Gsk3β mediated by miR-410 increased the expression levels of total Akt, total β-catenin, Oct4, Sox2, Nanog and CXCR4 whereas decreased the expression levels of phosph-Akt and phosph-β-catenin (Ser33/37/Thr41). The levels of miR-410 and Gsk3β might be correlated to clinicopathological differentiation in NSCLC tumor specimens.

Oct4, Sox2 and Nanog were proposed to be critical markers for lung cancer stem cells [29–33]. In this study, we demonstrated the expressions of Oct4, Sox2 and Nanog were notably increased in miR-410 stable overexpressed cells versus that were significantly decreased in miR-410 stable knock-down cells. And, *in vitro* and *in vivo* experiments showed stem cells capacities (such as sphere formation, metastasis, proliferation and chemoresistance) were increased in miR-410 overexpression stable cells versus those capacities were decreased in miR-410 knock-down stable cells. These results indicated miR-410 promoted stem cells-like features via up-regulation of Oct4, Sox2 and Nanog in NSCLC. CXCR4 was newly acknowledged as stem cells surface marker in several of tumors (including glioma, pancreatic adenocarcinoma and synovial sarcoma) [34–36]. Upregulation of CXCR4 was functionally crucial for maintaining stemness in gefitinib-resistant NSCLC A549 cells [37]. The inhibition of CXCR4 restrained stem cells-like properties (metastasis and chemoresistance) of CD133^+^/CXCR4^+^ stem cells derived from human NSCLC tumor tissues [38]. In this study, we found CXCR4 was obviously increased in miR-410 overexpression stable cells. Therefore, we inferred that CXCR4 might also participate in stemness induction by miR-410 in NSCLC. However, it still needed to be further studied.

CD44 and CD166 were reported to be stem cells surface markers of lung cancer in several researches [24–27]. Our results showed both CD44 and CD166 were apparently upregulated in miR-410 overexpression stable cells versus those were downregulated in miR-410 knock-down stable cells. These results also indirectly proved that miR-410 increased stemness of NSCLC cells.

What's more, we demonstrated that miR-410 directly targeted Gsk3β and inhibited its expression post-transcriptionally. In tumor tissues, miR-410^high^Gsk3β^low^ expressions were frequently found in low-differentiated tumor tissues while miR-410^low^Gsk3β^high^ expressions were frequently found in moderate-differentiated tumor tissues. These results demonstrated that miR-410 expression might be correlated to clinicopathological differentiation in tumor tissues. Interestingly, we found that expression levels of miR-410 were not statistically correlated to metastasis in tumor tissues. It was conflicting with the results in lung cancer cells lines. We thought that more tumor specimens might be needed to make such a statistical difference in the future.

It was reported that the inhibition of Gsk3β could increase the sensitivity to gemcitabine in pancreatic cancer PANC-1 cells [39]. In head and neck cancers, the inhibition of Gsk3β also reduced the expressions of stem cells markers such as Oct4, Sox2, and Nanog, but increased the levels of differentiation markers Calgranulin B and Involucrin in CD44^high^/ESA^high^ cells fraction [40]. It indicated that Gsk3β was involved in determining and maintaining stemness of CSCs in head and neck cancer. Gsk3β played a similar role in prostatic cancer [41]. These reports were contradictory with our results that miR-410 induced stemness by inhibiting Gsk3β. However, in lung cancer, there were also other reports which were in consistent with our results. It was reported that inhibition of Gsk3β increased the resistance to cisplatin in cisplatin-resistant A549 cells and this process was correlated to activation of Wnt/β-catenin pathway [42]. MiR-554a maintained self-renewal of lung cancer stem cells via downregulating Gsk3β [43]. We explained this contradiction was due to its diverse roles in distinct tumors.

As to in NSCLC, the role of Wnt/β-catenin signaling was still obscure in regulating stemness. We newly reported miR-410 promoted the progression of NSCLC which was correlated to Wnt/β-catenin pathway [16]. Herein, we further revealed miR-410 activated Wnt/β-catenin pathway through directly targeted Gsk3β. And the upregulated expressions of stem cells markers such as Oct4, Sox2, Nanog and CXCR4 were mediated by miR-410/Gsk3β/β-catenin signaling axis. Overexpression of Gsk3β in miR-410 overexpression stable cells partly decreased the expression of total β-catenin, Oct4, Sox2, Nanog and CXCR4, but increased the expression of phosph-β-catenin (Ser33/37/Thr41). Similarly, inhibition of Gsk3β in miR-410 knock-down stable cells partly increased the expression of total β-catenin, Oct4, Sox2, Nanog and CXCR4, but decreased the expression of phosph-β-catenin (Ser33/37/Thr41). These results further identified miR-410 induced stemness via miR-410/Gsk3β/β-catenin signaling axis in NSCLC.

As was known that Akt worked in the upstream of Gsk3β and Gsk3β inactivated by Akt at a serine 9 phosphorylation caused an accumulation of β-catenin in the cytoplasm, as a result of facilitating the translocation of β-catenin to the nucleus in which it activated the expressions of downstream targets [28], we made an attempt to explore whether inhibition of Gsk3β by miR-410 was due to the activating of Akt. Our results showed the protein levels of total Akt and phosph-Gsk3β (Ser9) were increased but that of phosph-Akt was decreased in miR-410 overexpression stable cells. Meanwhile, the protein levels of total Akt and phosph-Gsk3β (Ser9) were decreased and that of phosph-Akt was increased in miR-410 knock-down stable cells compared with their respective NC control cells. These results indicated miR-410 could also inhibit Gsk3β expression by increasing the expression of Akt except for directly targeting the 3′UTR of Gsk3β. However, the in-depth signaling pathway network among miR-410, Akt and Gsk3β needed to be further studied. In conclusion, we firstly revealed a novel mechanism that miR-410 could promote the tumorigenesis and development via inducing stemness in NSCLC.

## MATERIALS AND METHODS

### MicroRNA target prediction

The miRNA targets predicted by publicly available algorithms were obtained from miRanda (http://www.microrna.org/microrna/home.do), TargetScan (http://www.targetscan.org) and miRDB (http://www.mirdb.org/miRDB/). Putative target genes predicted by three algorithms were selected as candidates.

### Cells lines and clinical tissues

A549, H1299 and HEK293T cells lines were obtained from American Type Culture Collection (USA). The A549 and H1299 cells were cultured in RPMI 1640 (Invitrogen, Carslabd/CA, USA), and HEK293T cells were cultured in dulbecco's modified eagle medium (DMEM) (Invitrogen, Carslabd/CA, USA) supplemented with 10% fetal bovine serum (Invitrogen, Carslabd/CA, USA) at 37°C in 5% CO_2_.

Human NSCLC tumor and adjacent non-tumorous tissue samples were obtained from Department of Thoracic Surgery, West China Hospital, Sichuan University. This study was performed with the approval of the Medical Ethical Committee of West China Hospital, Sichuan University. A summary of the patients and tumorous samples characteristics are shown in Table 1.

### Quantitative real-time PCR

Total RNAs isolated from clinical tissues or cells lines using TRIzol Reagent (Invitrogen) were reversely transcribed to cDNA using a PrimeScript™ RT-PCR Kit (Takara Biotech (Dalian) Co., Ltd, Dalian, China). QRT-PCR was performed using a SYBR Green Real-time PCR Master Mix Kit (Bio-Rad, Hercules/CA, USA) on CFX96 Real-Time System (Bio-Rad, Hercules/CA, USA). β-actin and U6 were used as internal controls for gene and miRNA respectively. All reactions were performed in triplicate, and the relative expressions of miRNA or gene were calculated using the 2^−ΔΔCt^ method.

### Lentivirus infection and establishment of stable cells lines

MiR-410 overexpression, inhibition and scrambled control lentivirus solutions were purchased from GenePharma (Shanghai GenePharma Co. Ltd, Shanghai, China). MiR-410 and its interfered siRNA sequences were as follows: miR-410, AAUAUAAC ACAGAUGGCCUGU; miR-410-siRNA, UUAUAUUGU GUCUACCGGACA. Briefly, A549 or H1299 cells cultured in 24-well plates were infected with lentivirus particles or scrambled control clone with Polybrene (5 μg/ml; Sigma, St.Louis, MO, USA). Medium containing lentivirus particles was replaced with fresh medium 24 h post-infection. Stable cells were selected after 72 h infection using puromycin (2 μg/ml; Roche, USA) by 3–4 weeks. The stable cells lines were further identified by detecting miR-410 expression by qRT-PCR. Antibiotic-resistant cells were pooled for subsequent analysis.

### Cells proliferation and colony formation assay

For viable cells quantification, miR-410 over- expression or knock-down stable A549 cells and miR-410 overexpression or knock-down stable H1299 cells were seeded onto 96-well plates (3000/well). Cells viability was evaluated with 3-(4, 5-dimethylthiazol-2-yl) -2, 5-diphenyltetrazolium bromide (MTT; Sigma, St. Louis, MO, USA) as described previously [16].

For colony formation assay, miR-410 overexpression or knock-down stable A549 cells and miR-410 overexpression or knock-down stable H1299 cells were plated into 6-well dishes (1000/well) and cultured for 7 days. Cells colonies stained with 0.1% crystal violet were photographed, and those colonies with a diameter larger than 50 μm were counted.

### Boyden cells transwell and millicells assay

*In vitro* Transwell and Millicells assay were used for detecting the influence of miR-410 on invasion and migration of A549 and H1299 cells. For the migration assays, 1 × 10^5^ miR-410 overexpression or knock-down stable A549 cells and 1 × 10^5^ miR-410 overexpression or knock-down stable H1299 cells in serum-free media were placed respectively into the upper chamber of an insert (8-μm pore size, Millipore, Billerica, MA, USA). For the invasion assays, 3 × 10^4^ miR-410 overexpression or knock-down stable A549 cells and 3 × 10^4^ miR-410 overexpression or knock-down stable H1299 cells were placed into the upper chamber of an insert coated with Matrigel (BD Biosciences, San Diego, CA, USA). Media containing 10% FBS were added to the lower chamber. After 24 hours of incubation, the cells remaining on the upper membrane were removed with cotton wool, whereas the cells that had migrated or invaded through the membrane were stained with methanol and 0.1% crystal violet, imaged and counted.

### Drug resistance

MiR-410 overexpression or knock-down stable A549 cells and miR-410 overexpression or knock-down stable H1299 cells cultured in the 96-well plates were treated with cisplatin (0, 2, 4, 8, 16, 32, 64 μg/ml). 24 h after treatment, viable cells were evaluated with 3-(4, 5-dimethylthiazol-2-yl) −2, 5− diphenyltetrazolium bromide (MTT; Sigma, St. Louis, MO, USA) as described previously [44]. IC_50_ was analyzed by SPSS 19.0 software.

### Sphere formation assay

To assay sphere formation, miR-410 overexpression or knock-down stable A549 cells and miR-410 overexpression or knock-down stable H1299 cells were plated in ultra-negative attachment 6-well plates (Corning) at a density of 6000 viable cells/well [45]. Cells were grown in a serum-free sphere culture medium DMEM/F12 supplemented with N-2 supplement, 10 ng/ml EGF, 20 ng/ml IGF and 10 ng/ml bFGF (Invitrogen). After 9 days of culture, the numbers of tumor spheres formed were counted using an inverted microscope.

### Western blotting and immunofluorescence staining

Western blotting analysis was performed as described previously [22]. The following primary antibodies were used: anti-Nanog, anti-Sox2, anti-Oct4, anti-β-catenin, anti-phospho β-catenin (Ser33/37/Thr41), anti-Gsk3β, anti-phospho Gsk3β (Ser9), anti-Akt, anti-phospho Akt, anti-β-actin (Cells Signaling Technology, Danvers, MA, USA, 1:1000), anti-CXCR4 (Abcam, USA, 1:2000). β-actin was used as an internal control.

For immunofluorescence staining, miR-410 overexpression or knock-down stable A549 cells and miR-410 overexpression or knock-down stable H1299 cells were seeded onto poly-l-lysine coated glass coverslips in 6-well dishes. After 24 h, the cells were fixed with 4% formaldehyde in PBS, permeabilized in PBS containing 0.5% Triton X-100 and blocked with 1% bovine serum albumin in PBS. Then cells were incubated with anti-Gsk3β (Cells Signaling Technology, Danvers, MA, USA, 1:200) antibody overnight. The next day, cells were washed and incubated with TRITC-conjugated anti-mouse or anti-rabbit antibody (ZhongShanJinQiao Biotechnology, Beijng, China, 1:100) or DAPI (4′, 6-diamidino-2- phenylindole).

### Plasmid construction and siRNAs

The wild-type and mutant 3′UTR sequences of Gsk3β were cloned into the pmirGLO vector (Promega (Beijing) Biotech Co., Ltd, Beijing, China) respectively, and confirmed by sequencing. Gsk3β cDNA (NM_001146156) were cloned into pVax vector to construct its expression plasmid and confirmed by sequencing. To interfere the expression of Gsk3β, three siRNAs were designed (1#, UUUAGUGUCUGUAUAGCUGTT; 2#, AUUCUUAAAUCUCUUGUCCTT; 3#, AGUUGGUGU AUACUCCAGCTT).

### Luciferase reporter assay

Dual-Luciferase Reporter System (Promega (Beijing) Biotech Co., Ltd, Beijing, China) was used to analysis whether miR-410 could directly target the 3′UTR of Gsk3β. For luciferase reporter assay, miR-410 overexpression stable cells in 96-well plates were transfected with pmirGLO-Gsk3β-3′UTR -wild (3-UTR-W) or pmirGLO-Gsk3β-3′UTR-mutant (3-UTR-M) or pmirGLO plasmids following the manufacturer's protocol of Lipo2000 (Invitrogen, Carslabd/CA, USA). Luciferase activity was detected 24 h post-transfection according to dual-luciferase reporter assay system (Promega (Beijing) Biotech Co., Ltd, Beijing, China). The Renilla luciferase signals were normalized to the internal firefly luciferase transfection control. Transfections were done at least thrice in independent experiments.

### Subcutaneous lung tumor and lung metastasis model, H&E staining and immunohistochemistry

3–4 weeks old female athymic BALB/c nude mice (BEIJING HFK BIOSCIENCE CO. LTD, Beijing, China) were maintained in a specific pathogen-free (SPF) environment. All studies on mice were admitted by the National Institutes of Health ‘Guide for the Care and Use of Laboratory Animals’.

In order to detect the effects of miR-410 on tumorigenicity and metastasis of NSCLC *in vivo*, miR-410 overexpression or knock-down stable A549 cells and negative control cells were used to establish subcutaneous lung tumor and lung metastasis mouse model respectively according to the described method [44, 46].

For subcutaneous lung tumor mouse model, miR-410 stable overexpressed or knock-down A549 cells were subcutaneously injected into nude mice (5 × 10^6^ cells per mouse, seven mice in each group). The effective tumor volume was calculated when it reached about 100 mm^3^ (about 4 weeks). Eight weeks post inoculation (The volume of tumor tissues reached ~ 800 mm^3^ in group treated with miR-410 stable overexpressed cells), mice were anesthetized by ether inhalation and tumor tissues were harvested. For immunohistochemistry, the sections were treated with 3% H_2_O_2_ and incubated with anti-Gsk3β (Cells Signaling Technology, Danvers, MA, USA, 1:400), or anti-Ki-67 (Cells Signaling Technology, Danvers, MA, USA, 1:400) antibody as described previously [47].

For lung metastasis mouse model, miR-410 overexpression or knock-down stable A549 cells were injected via tail vein into nude mice (3 × 10^6^ cells per mouse, eight mice in each group). About eight weeks later, eight mice were anesthetized, and lungs of five mice were injected intratracheally with India ink and fixed by AAF solution to count the numbers of metastatic tumor nodules (white dots) on lung surfaces. The sizes of the metastatic nodules were observed and the relative metastatic ratio was calculated in terms of the tumor nodules in each group. Lungs of three mice fixed in 4% buffered paraformaldehyde were stained with H&E to visualize the metastatic tumor nodules as described previously [44].

### Statistical analysis

Statistical analysis was performed by SPSS 19.0 software. Data was presented as the means ± standard deviation (SD). All experiments were done in triplicate. The statistical significance of the results was calculated using one-way analysis of variance and an unpaired Student's *t*-test. *P* < 0.05 was supposed to be statistically significant.

## Abbreviations

NSCLC: non-small cells lung cancer
miRNA: microRNA
CSCs: cancer stem cells
CXCR4: chemokine receptor-4
sFRPs: secreted Frizzled -related proteins
QRT-PCR: quantitative real-time PCR
H&E: hematoxylin and eosin
